# Molecular Characterization of camphor utilizing bacterial isolates from refinery sludge and detection of target loci-Cytochrome P-450 _*cam*_ mono oxygenase (*cam* C gene) by PCR and gene probe

**DOI:** 10.1186/2193-1801-2-170

**Published:** 2013-04-17

**Authors:** Ganesan Bhuvaneswari

**Affiliations:** Environmental Genomics Unit, National Environmental Engineering Research Institute, Nehru Marg, Nagpur, 440 020 Maharashtra India; Seribiotech Research Laboratory, Carmelaram Post, CSB Campus, Kodathi, Bangalore, 560 035 Karnataka India

**Keywords:** Sludge sample, Camphor, 16S r DNA PCR, ARDRA, *cam* C gene PCR, Dot-blot, Phylogenetic analysis

## Abstract

This study presents the isolation and molecular characterization of bacterial strains utilizing 1, 7, 7-Trimethylbicyclo (2.2.1) heptane-2-one (camphor) as a sole source of carbon, isolated from the biomass sludge sample collected from an effluent treatment plant of Mathura Refinery Limited (MRL), India. Initial screening was carried out where the 16S rDNA PCR was performed using reported eubacterial primer set followed by Amplified Ribosomal DNA Restriction Analysis (ARDRA). About 47% of the isolates have shown unique ARDRA pattern based on which, 15 distinct isolates were selected and tested for the presence of *cam* C gene that was successfully demonstrated by PCR using gene specific primers. A Dot-Blot experiment was designed to detect the *cam* C loci in the plasmid DNA of all camphor isolates based on non-radioactive “Biotin-Streptavidin” detection system. The bacterial identity with respect to partial 16S rDNA gene sequences of all camphor isolates placed them in 9 major genera viz., *Pseudomonas* sp., *Staphylococcus* sp*., Alcaligenes* sp., *Agromyces* sp*., Stenotrophomonas* sp., *Reichenowia* sp., *Achromobacter* sp.*, Brevibacterium* sp. and *Pseudaminobacter* sp. A detailed phylogentic tree was also constructed to establish their evolutionary status from the gene sequence data.

**Electronic supplementary material:**

The online version of this article (doi:10.1186/2193-1801-2-170) contains supplementary material, which is available to authorized users.

## Background

Poly aromatic hydrocarbons (PAHs) are imposing serious threats to human health and are constantly affecting the surrounding environment due to its highly recalcitrant nature that reduces its bioavailability to natural degradation and leads to its prolonged existence in the environment. This has forced researchers to look out for efficient bioremediation strategies to degrade these hazardous pollutants. Microbial biodegradation is an efficient and promising solution to such environmental restoration (Stapleton and Sayler 1998). The application of available advanced molecular biology tools have led to the designing of rapid and accurate strategies to monitor, discover and identify novel bacteria and their catabolic potentialities with respect to degradation of several environmental xenobiotics such as PAHs (Ahn et al. 1999; Goyal and Zylstra 1996; Hedlund et al. 1999; Zylstra et al. 1997). The unbound limits of microbial ecology demonstrate that, different bacteria exist in same ecosystem having similar catabolic options for survival. The survival of bacteria using PAH as substrate has always remained a challenging area of research. With an increase in information of diverse genes that encode enzymes for PAH catabolism (Mueller et al. 1997; Saito et al. 1999), the next step is to understand the functions of these genes and also to determine their ecological significance in context of environmental pollution. Hence in-depth knowledge of PAH-catabolic genes from diverse groups of bacteria will provide valuable information pertaining to fundamentals of bacterial catabolic mechanisms and will aid in designing efficient bioremediation strategies (Shimada 2006). However there is a trial calculation stating, only 1% of bacteria that actually exist on earth have been isolated (Rodon et al. 1999). Therefore, a molecular approach with PCR and gene probes targeting important catabolic genes would be very useful for further detection and characterization of cultivable as well as uncultivable microbial degraders (Hamann et al. 1999; Meyer et al. 1999).

In our present study we have used sludge sample collected from the sewage outlets of the Mathura Refinery Limited (MRL). This refinery located at Mathura, was commissioned in the year 1982 as India’s sixth oil refinery. Refineries commonly face issues of environmental safety and concerns and have to strictly abide by the environmental protection regulations set by the Governmental agencies. However the various complex stages of refinery procedures are associated with inevitable elimination of large amount of chemicals especially the PAHs that pollute the surrounding. Earlier studies have reported presence of PAHs in refinery soils. Masih and Taneja (2006) have estimated the average concentration of PAHs in the soil samples collected from sites adjacent to Mathura Refinery ranging from 3.1 to 28.5 μg/g of soil. Yet another study revealed Fluoranthene, Chrysene and benzo fluoranthene as most abundant PAHs of this site (Rawat and Sharma 2008). The presence of heterotrophic microorganisms in soil act as significant biological factors, that allows these chemicals to be utilized as a carbon source. In other words the microbial flora is highly influenced by the type of pollutant persisting in the contaminated site. This in turn results in enhanced degradation capabilities by means of activated expression of key genes associated with catabolic enzymes as a vital step towards adaptation and enrichment of such microbial degraders. In this study we have targeted one such significant gene, the “Cytochrome P-450 cam mono oxygenase loci” also known as *cam* C gene with respect to the chosen substrate i.e., Camphor, that was used for enrichment of microbes present in the collected sludge sample. Earlier report by (Chakrabarty 1976), clearly states that the various genes coding for Cytochrome P-450 _cam_ and many other enzymes of the camphor degradative pathway reside in a 230 kbp CAM plasmid operon which constitutes the *camC* gene (M12546.1) loci. The complete nucleotide sequence of the *cam* C gene in *Pseudomonas putida* as reported by Unger et al. (1986) was successfully cloned and also expressed in *Escherichia coli*. The Cytochrome dependent mono oxygenases are known for their broad substrate specificity and play a pivotal role in catabolizing a series of PAHs, especially the low molecular weight PAHs like phenanthrene, naphthalene, anthracene, salicylate, catechol etc., which are known for years to be good substrates for bacteria (Harford-Cross et al. 2000; Kim et al. 2004). Since camphor is the natural substrate to this ubiquitous enzyme- Cytochrome P-450 cam mono oxygenase (Susanna et al. 2000), hence we used camphor as a sole source of carbon to isolate and characterize bacteria from the PAH contaminated sludge sample collected from the MRL sewage outlets.

## Results

### Selection of diverse camphor degrading bacteria based on 16S r DNA PCR and ARDRA

**Figure 1:**
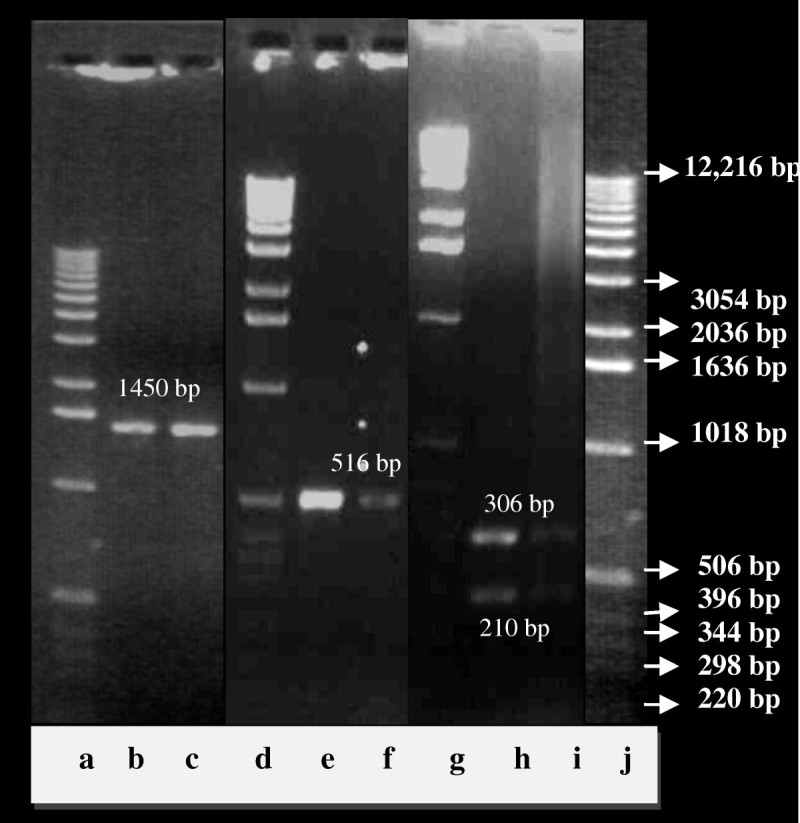
**(a-c) 16S rDNA gene PCR in camphor isolates.** Lanes: **a**- DNA ladder; **b** –positive control; c- camphor isolate. **(d-f)*cam*C** gene PCR in camphor isolates. Lanes: **d**- DNA ladder; **e**- positive control; **f**- camphor isolate. **(g-i)***Eco* RV Restriction digestion pattern of the *cam* C gene PCR amplicon. Lanes: **g**- DNA ladder; **h & i**- restriction digestion patterns of the *cam* C gene of positive control and camphor isolate showing two bands corresponding to 306 and 210 base pairs respectively. **(j)** Lane shows 1 kb DNA ladder from Gibco-BRL with detailed MW specifications. Note: Gel picture is divided in three segments, showing results of positive control and one test isolate only (representing remaining camphor isolates that are not included in the gel image).

**Figure 2:**
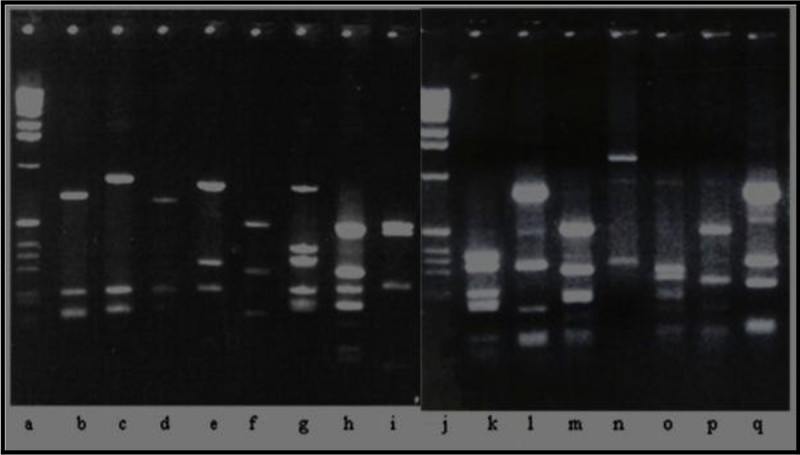
**ARDRA: Unique band pattern for the 15 camphor-utilizing bacteria obtained by restriction digestion of 16S r DNA PCR products of each isolate with*Hae*III restriction enzyme.** Lanes: **a** &**j-** 1.0 kb DNA ladder (Gibco-BRL); lanes **b**-**i** and **k**-**q** shows the unique digestion pattern of the 16S ribosomal DNA with respect to the different camphor isolate.

This is a preliminary report on a diverse group of bacteria isolated from the MRL sludge that were fed with camphor as a sole source of carbon. The cultures were enriched twice with 100 ppm of camphor in the minimal media to selectively isolate strains efficiently using camphor as a substrate. Initially about 32 distinct bacterial colonies were selected based on their basic morphological features such as shape, size and color. The PCR amplification of 16S r DNA gene was done using the Universal Eubacterial primer set 27 F and 1492 R. All bacterial isolates have shown successful amplification of 1450 bp PCR amplicon of 16S r DNA gene (Figure 1, lanes a-c). As a follow-up step, 16S r DNA PCR amplicons were subjected to ARDRA using restriction enzyme *Hae* III. Digestion analysis revealed that, out of 32 test isolates, about 15 isolates were showing discrete fragmentation pattern accounting to 47% of the total camphor utilizing HPC strains isolated in this study. The unique ARDRA patterns for the final 15 camphor isolates are shown in Figure 2.

### PCR amplification of target cam C gene and its detection in camphor isolates using gene specific probe

**Figure 3:**
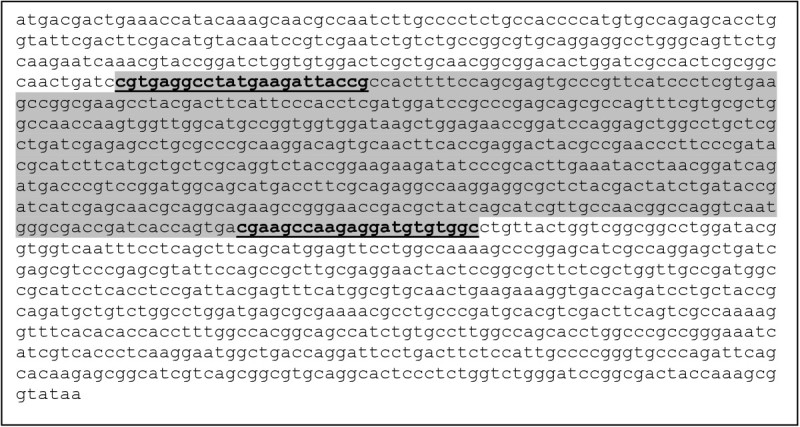
***Pseudomonas putida- cam*C gene, encoding Cytochrome P-450-cam, complete cds.** AC#M12546. Unger et al. (1986). Gene sequence amplified by PCR (516 bp) has been highlighted. Primer sequences are underlined.

The colony PCR was carried out for all the 15 isolates targeting the *cam* C gene. The primers were designed with reference to the conserved regions of *cam* C gene of *Pseudomonas putida* (Unger et al., 1986, AC# M12546) as shown in Figure 3. Hence, *P. putida* standard strain was taken as a positive control in PCR reaction. All strains showed successful amplification of the gene with expected size of 516 base pairs (Figure 1, lanes d-f). The authenticity of the amplified *cam* C PCR product was further confirmed by restriction digestion analysis using *Eco* RV with reference to the restriction map of the cam C gene of *P. putida*. The restriction pattern of the *cam* C gene product from the isolates was compared to that of the positive control as shown in Figure 1 (lanes g-i).

**Figure 4:**
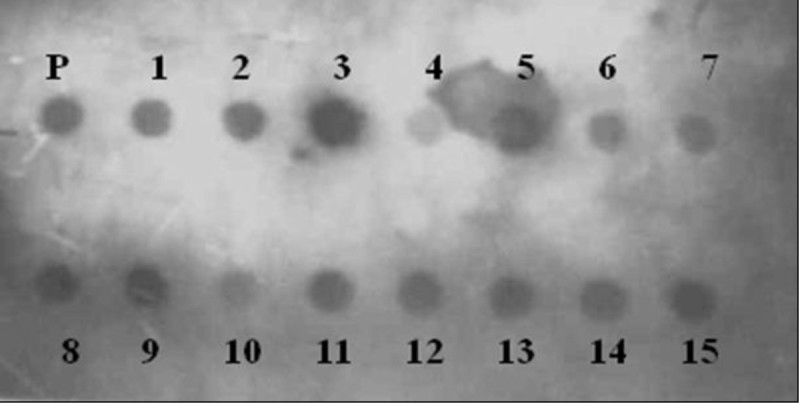
**DNA dot-blot experiment: detection of*cam*C gene in from the Plasmid DNA of camphor isolates.** P – Plasmid DNA of positive control ; 1 to 15 shows positive signal with respect to the presence of *cam* C gene in Plasmid DNA of all 15 unique camphor utilizing HPC-isolates.

In this study we further did the DNA dot-blot experiment for all camphor isolates (Figure 4) by spotting their Plasmid DNA along with the positive control onto the N-Bond Nylon membrane using the Dot-Blot apparatus. The *cam* C PCR product of the positive control was used for generating the biotinylated probe that was conjugated to SA-AP during the blotting steps. The detection of probe, bound to the target site was successfully demonstrated, based on the chemiluminescence reaction between the Streptavidin linked alkaline phosphatase and the chemiluminescent substrate–Lumiphos, where the final detection signals were captured in a photographic film.

### Identification of bacterial strains and their Phylogenetic relationships

In order to establish the identity of 15 camphor isolates, the 16S r DNA PCR products were cloned, sequenced and submitted to GenBank of the NCBI and were matched with the available sequences in database. The BLAST search results have placed the camphor isolates belonging to 9 major genera viz., *Pseudomonas* sp. (Isolates_HPC-319, HPC-326 and HPC-330)*, Staphylococcus* sp. (Isolates_HPC-322, HPC-323 and HPC-324)*, Alcaligenes* sp. (Isolates_HPC-328 and HPC-333), *Stenotrophomonas* sp. (Isolates_HPC-320 and HPC-329), *Brevibacterium* sp. (HPC-331), *Reichenowia*, sp. (HPC-332), *Agromyces* sp. (HPC-334), *Achromobacter* sp. (HPC-325) and *Pseudaminobacter* sp. (HPC-321)*.* The GenBank Accession numbers for all 15 camphor utilizing HPC- strains along with their identity are mentioned in Table 1.

**Table 1:** **Details of strain identity along with their accession numbers as submitted in NCBI GenBank for the 15 camphor utilizing HPC strains**

***Isolate***	***GenBank accession No.***	***Bacterial strain***
HPC-319	AY897403	*Pseudomonas putida strain*
HPC-320	AY897404	*Stenotrophomonas acidaminiphila*
HPC-321	AY897405	*Pseudaminobacter* sp.
HPC-322	AY897406	*Staphylococcus aureus strain*
HPC-323	AY897407	*Staphylococcus aureus strain*
HPC-324	AY897408	*Staphylococcus epidermis*
HPC-325	AY897409	*Achromobacter xylosoxidans*
HPC-326	AY897410	*Pseudomonas* sp.
HPC-328	AY897412	*Alcaligenes* sp.
HPC-329	AY897413	*Stenotrophomonas* sp.
HPC-330	AY897414	*Pseudomonas* sp.
HPC-331	AY897415	*Brevibacterium* sp.
HPC-332	AY897416	*Reichenowia* sp.
HPC-333	AY897417	*Alcaligenes* sp.
HPC-334	AY897418	*Agromyces* sp.

**Figure 5:**
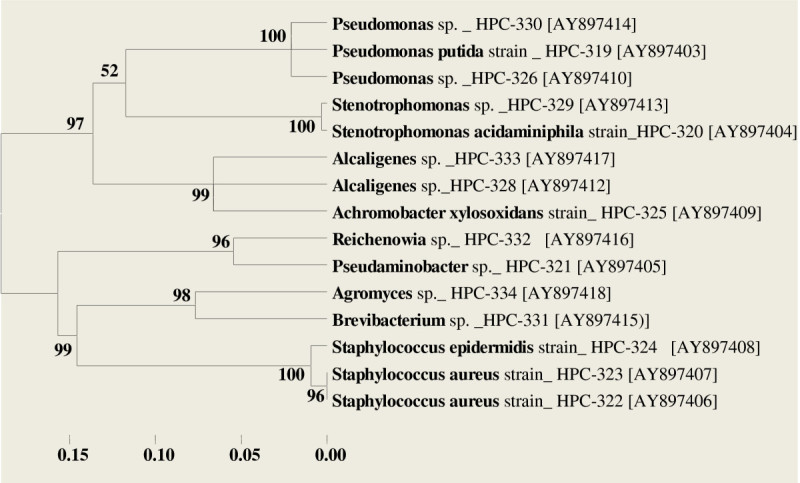
**Phylogenetic tree for the 15 camphor utilizing HPC-Strains based on their partial 16S r DNA gene sequences.** Dendrogram generated using CLUSTAL-MUSCLE for sequence analysis and MEGA-5 for tree construction using Neighbor Joining methods. Boot strap values for clustered taxa are mentioned at the tree branch points (500 replicates taken).

As a conclusive step to our study, the phylogenetic inference was drawn. A dendrogram was generated based on the partial 16S r DNA nucleotide sequences of all test isolates. The sequence alignment was done using CLUSTAL-MUSCLE program clubbed with the latest 5^th^ version of software-MEGA for generation of tree, as shown in Figure 5. We have used the Neighbor joining Algorithm (NJ) method. All positions containing gaps and missing data were eliminated from the data set by complete deletion option. The percentage of replication trees in which the taxa clustered together in bootstrap test (500 replicates) is shown next to branch point.

## Discussion

Our lab has previously reported on isolation of bacteria from environmental niches degrading phenolics (Kutty et al. 2000, 2001; Narde et al. 2004; Qureshi et al. 2001, Qureshi and Purohit 2002). Refinery sludge is a challenging waste, highly polluted with PAHs. The present study focuses on diverse bacterial population surviving in such environments, capable of utilizing these hazardous chemicals as carbon source. Hence, the target is looking for specific catabolic genes present in these microbes, that can be applied in near future for designing intrinsic bioremediation strategies of the polluted sites.

Cytochrome P450 monooxygenases (CYPs or P450s) are from ubiquitous protein family existing in all eukaryotes, and in most of the prokaryotes and Archae. These heme containing enzymes are versatile biocatalysts, involving monooxygenation of large variety of substrates and therefore the discovery of new P450s with novel activities from diverse organisms is being focused for potential biotechnological applications. In a recent review, Urlacher and Girhard (2012) have presented an overview on recent advancements with respect to P450 engineering for synthetic applications such as drug development, bioremediation and production of fine chemicals. In our study we have targeted the Cytochrome P450 _cam_ monooxygenase gene (*cam* C) in the isolated strains.

The widely accepted PCR based 16S rDNA gene analysis (Weisburg et al. 1991), was used for bacterial identification and Phylogenetic studies, since it is highly conserved on evolutionary scale, yet diverse enough to identify and classify Eubacteria (Purohit et al. 2003). The 16S rDNA gene PCR products were subjected to ARDRA technique as reported by Gurtler et al. (1991). This has been proved to be a powerful tool to identify different polymorphic groups among the amplicons that in turn reflects the differences within the sequence of the 16S r RNA gene and therefore allows the inference on the phylogenetic relationships among taxonomically, relatively close and distant organisms reflecting Eubacterial diversity (Ludwig et al. 1998). In addition, novel or rare microorganisms could also be identified on the basis of unique sequences and digestion patterns. Based on this principle, ARDRA has also been used as a diagnostic tool (Backermans and Madsen 2002).

All the diverse isolates selected by 16S rDNA and ARDRA analysis were further screened for the presence of target gene of our interest i.e., *cam* C gene, that was successfully amplified in all the test isolates. Based on the the restriction map of reference gene, *Eco* RV was chosen for restriction analysis as a step towards establishing sequence authenticity. Since, it has a single restriction site at position 306 bps, thereby cleaves the gene product into two fragments corresponding to molecular sizes of 306 bps and 210 bps respectively. An exact match of the digested pattern of cam C gene amplicon of all test isolates with respect to that of the positive control was established, which confirmed the correctness in *cam* C gene sequence of the studied isolates.

The detection of target loci *cam* C using gene probe was based on the popular nonradioactive, chemiluminescent and extremely sensitive approach of the Biotin-Streptavidin system (Diamandis and Christopoulos 1991). Our Dot-Blot experiment has successfully demonstrated the positive binding of *cam* C gene probe to the spotted plasmid DNA from all 15 camphor isolates along with the positive control, which suggests the localization of *cam* C gene to the plasmid DNA. This result is in consistence with the earlier report by Shaham et al. (1973) who clearly described about the camphor utilizing strains of *P. putida* carrying the genetic information required for camphor degradation on a plasmid.

The sequencing of the 16S r DNA from the isolates revealed that, most of the bacterial strains were members of the *Proteobacteria* (*α-, β-, γ- Proteobacteria*) class. Among the 15 strains isolated from the sludge sample the predominant group was γ*- Proteobacteria* (*Pseudomonas* sp., *Stenotrophomonas* sp*.*). Isolates belonging to *α- Proteobacteria* (*Reichenowia* sp., *Pseudaminobacter* sp.) were also present. Representatives of *β-Proteobacteria* (*Alkaligenes* sp., *Achromobacter xylosoxidans*) were also found beside members of classes *Actinobacteria* (*Agromyces* sp., *Brevibacterium* sp.) and *Fermicutus* (*Staphylococcus aureus* strain and *Staphylococcus epidermis*). Interestingly we have isolated three strains belonging to the genera of *Staphylococcus* sp*.* These strains are generally related to skin and respiratory infections in human and are rarely associated with PAH degradation. However there have been a few relevant reports before. Survery et al. (2004) have reported strains of *Staphylococcus* sp. isolated from soil near to petrol pumps in Karachi city, Pakistan, that were able to degrade base oil. Yet another interesting report by Mallick et al. (2007) states about a *Staphylococcus* sp. strain PN/Y capable of degrading Phenanthrene as a sole source of carbon. This strain was isolated from the petroleum contaminated soil of Noonmati Refinery site, India and was found to carry a mega plasmid-p PHN, approximately of 112 kb size, that was responsible for the degradation of Phenanthrene by a novel assimilation pathway. Kafilzadeh et al. (2011) have also reported bacterial strains belonging to *Staphylococcus* sp. isolated from contaminated soils of Iran, which were able to degrade naphthalene. So far, plasmid mediated biodegradation of PAHs have been widely reported in both Gram positive and Gram negative bacteria, but very little is known about the hydrocarbon degradation capability by genus *Staphylococcus* sp. The acquisition of catabolic ability mediated through plasmids in such unusual bacterial hosts has been attributed to two possible mechanisms of horizontal gene transfer and recombination by transpositions that are closely linked (Obayori and Salam 2010) and thus plays a vital role in acclimatization of bacterial communities to various pollutants. Thus in our study, we predict that horizontal transfer of *cam* C gene may have occurred in *Staphylococcus* sp. strains from the surrounding bacterial population resulting in camphor uptake and its utilization.

Earlier report by Kumar and Khanna (2010), who determined the bacterial community structure of a coal-tar contaminated soil by establishing a clone library of 16S r RNA genes, had clearly stated that most of the hydrocarbon polluted sites are inhabited with *Proteobacteria* consortia which majorly constitute the *Gammaproteobacteria* and *Actinobacteria*. Our findings confirm this report.

## Conclusions

Biodegradation of organic chemical pollutants is one of the many important processes affected in field sites by microorganisms which use them as a source of carbon and energy. The predominance and persistence of a particular type of pollutant also shifts the microbial communities towards those organisms which can utilize these contaminants and survive. Hence they also act as an indicator species to monitor specific pollutants on site. Thus in this study we have attempted to isolate a diverse group of bacterial strains belonging to various genera from a PAH contaminated sludge sample collected from a refinery site using camphor as a carbon source and tried to characterize them at a phylogenetic level in order to check their diversity. The localization of target loci, Cytochrome P-450 camphor mono oxygenase gene in host plasmid was successfully demonstrated by PCR and gene probe methods. Microbial communities are promising source for diverse catabolic potentialities which should be harnessed by human to generate novel bioremediation strategies to clean up this heavily polluted environment.

## Methods

### Chemicals

All the growth media components were purchased from Sigma Chemical Co. (St. Louis, USA). *Taq* DNA polymerase (Bangalore Genei-Merck), Agarose (USB) and 1 kb DNA ladder (Gibco-BRL) were used. Restriction enzymes (*Hae* III and *Eco* RV), N-Bond nylon membrane for Dot-Blot were procured from Amersham-GE Healthcare. Components for Hybridization buffer and blocking solution were procured from Sigma Chemical Co. (St. Louis, USA). BioPrime DNA Labelling System for probe generation and TA-Cloning Kit were from Invitrogen. Lumiphos (Chemiluminescent dye) was procured from Thermo Scientific.

### Growth media

The media employed in the bio-reactor was 0.1X M9 Media, containing (per liter) 20 ml of 5X Salts (Na_2_HPO_4_ - 64 g l^-1^, KH_2_PO_4_ -150 g l^-1^, NaCl - 2.0 g l^-1^), 200 μl of 1 M MgSO_4_ and 4 ml of 1 M Cacl_2_. The inoculum from the reactor sample was enriched in New Mineral Media (NMM) containing (per liter) 1 ml of 10%CaCl_2_, 2 ml of 10% MgSO_4_, 2.5 ml of 10% NH_4_Cl, 40 ml of 50 mM Phosphate buffer (Na_2_HPO_4_- 131 g l^-1^ and KH_2_PO_4_-67.5 g l^-1^).

### Enrichment and culturing steps

The MRL sludge was initially fed into bioreactor containing 0.1X M9 Media along with 250-ppm of crude oil as a substrate for about a month. One ml of treated sludge from the bioreactor was pelleted and washed twice with double distilled water. This pellet was then inoculated in NMM containing 100 ppm camphor, as the sole carbon source. The culture flask was enriched after five days by transferring the pellet into fresh NMM media along with camphor. Enrichment was done twice and was followed by serial dilution and spread plating on NMM plates containing camphor in order to isolate bacteria selectively utilizing camphor for their growth. The concentration of camphor in all enrichment steps was constantly maintained at 100 ppm.

### Template preparation for PCR amplification

Morphologically distinct bacterial colonies from the NMM plates were chosen for further molecular studies. They were picked up carefully on the tip of a sterile platinum inoculating needle and were inoculated in PCR tube containing 10 μl of PCR grade water. The cells were lysed by heating to 95°C for 5 minutes in PTC-200 thermal cycler (MJ Research) as described earlier by Park et al. (2002). About 5 μl of the denatured cells were taken for the PCR amplification of desired gene.

### PCR Amplification of 16S r DNA & Amplified Ribosomal DNA Restriction Analysis (ARDRA)

The PCR amplification of 16S rDNA gene for all the morphologically distinct camphor isolates was carried out as done earlier (Bhuvaneswari et al., 2005), using the eubacterial primer set- 27 F(5^′^-AGAGTTTGATCMTGGCTCAG-3^′^) and 1492 R (5^′^-TACGGYTACCTTGTTACGACTT-3^′^) as reported by Backermans and Madsen (2002). 5 μl of denatured cells of different isolates were used as a template in the PCR. The forward and reverse primers were used at a concentration of 50 pmol and the annealing temperature was set at 55°C. The resulting 16S rDNA PCR amplicons were confirmed by analyzing PCR them on 1.2% agarose gels containing 0.5 μg/ml of ethidium bromide in 1X TAE running buffer. The PCR products were concentrated by ethanol precipitation and were subjected to ARDRA using the restriction enzyme *Hae* III as per manufacturer’s protocol. The restriction band patterns were viewed in 1.8% agarose gel stained with ethidium bromide. Isolates with entirely discrete restriction pattern were chosen for further studies targeting the *cam* C gene.

### PCR amplification of cam C gene

PCR amplification for *cam* C gene was performed in a total volume of 50 μl. The reaction mixtures contained 2 μl of 10X-reaction buffer, 1.2 μl of 25 mM Mgcl_2,_ 2 μl of 2 mM d NTPs, 0. 5 μl of each primer viz., FP 5^′^-CGT GAG GCC TAT GAA GAT TAC CG-3^′^ and RP 5^′^-GCC ACA CAT CCT CTT GGC TTC G-3^′^ (GENBANK AC# M12546), 2U of *Taq* DNA Polymerase. About 5 μl of the denatured cells were used as template in the PCR. A cycling regime with initial denaturation at 94°C for 5 min (1 cycle), followed by 94°C for 1 min, 65°C for 1.5 min, 72°C for 2 min (35 cycles) and final extension at 72°C for 7 min (1 cycle) was employed. The successful amplification of *cam* C gene was confirmed by analyzing PCR products on 1.2% agarose gels as described above.

### Restriction analysis of cam C gene PCR amplicons

The *cam* C gene amplicons were concentrated using ethanol precipitation before carrying out restriction digestion. In ethanol precipitation the *cam* C PCR product were mixed gently with one-tenth volume of 3 m M sodium acetate (pH-5.2) and 2.5 volumes of absolute ethanol. This mix was incubated at minus 80°C for an hour and then centrifuged at 14,000 rpm for half an hour. The pellets obtained were dissolved in PCR grade water and were digested with 15U of *Eco* RV. The restriction digestion was carried out for 3 h at 37°C and the fragmentation pattern was viewed in 2% agarose gel stained with ethidium bromide.

### DNA Dot-blot

All the 15 isolates were recultured in NMM broth containing 100 ppm of camphor and were used for Plasmid DNA extraction using the conventional method (Sambrook et al. 1989). The Plasmid DNA of 15 camphor isolates along with one positive control was spotted onto the N-Bond nylon membrane using the Dot-Blot apparatus. The DNA spots on the membrane were denatured with 0.5 N NaOH and 1 M Tris for 5–6 min, one after the other. The membrane was then exposed for UV cross-link for 3 min followed by Pre-hybridization at 65°C for 1 hr. Hybridization was done at 60°C for 18 hrs after the addition of 50 ng of biotinylated *cam* C probe that was made using the BioPrime DNA Labelling System following the kit protocol. Blot washing steps included preliminary wash with 5X SSC and 0.5% SDS at 65°C followed by wash with 0.1X SSC and 1% SDS at 50°C. Brief washes with 2X SSC and TBS-Tween20 were done at room temperature. Blocking of the membrane was done with prewarmed (65°C) blocking solution (3 gm BSA/100 ml of TBS-Tween20) for 1 hr. The membrane was treated with 2000 fold diluted SA-AP (Streptavidin alkaline phosphatase) in TBS-tween 20 for 10 min at room temperature. Finally the membrane was washed for an hour at room temperature with final wash buffer composed of 100 mM Tris (pH-9.5), 100 mM NaCl and 50 mM of magnesium chloride. For chemiluminescent detection process, the dye namely 2-amino-2-methyl-1 propanol or Lumiphos was used at a concentration of 10 μl / square cm. The photographic film was developed after 15 min of exposure.

### Cloning and sequencing of 16S rDNA PCR amplicons of camphor isolates

The 16S-rDNA PCR product of 15 distinct camphor-utilizing bacteria was ligated into pCR 2.1-TOPO (TOPO TA-Cloning Kit; Invitrogen), following the manufacturer’s recommended protocol. Selection of clones was based on the conventional blue white screening using IPTG and X-Gal. The plasmids from all positive clones were extracted according to the standard protocol (Sambrook et al. 1989) and checked for the presence of right insert by viewing them in 1% Agarose gel. The positive recombinant plasmids carrying the 16S r DNA insert were column purified and sequenced.

### Phylogenetic analysis of the camphor isolates based on partial 16 S rDNA sequences

The partial 16S rDNA gene sequences for all the 15 camphor isolates were analyzed by matching them with the available sequence data in the NCBI database using BLAST and were later submitted in GenBank. The 16S rDNA nucleotide sequences of the 15 isolates were aligned using the CLUSTAL MUSCLE program as described earlier by Edgar 2004, and was used to generate the phylogenetic tree based on Molecular evolutionary genetic analysis (MEGA-5) software using the Neighbor joining method (Tamura et al. 2011).

## Authors’ original submitted files for images

Below are the links to the authors’ original submitted files for images. Authors’ original file for figure 1 Authors’ original file for figure 2 Authors’ original file for figure 3 Authors’ original file for figure 4 Authors’ original file for figure 5
