# Supplementary figures and images for: Molecular Characterization of camphor utilizing bacterial isolates from refinery sludge and detection of target loci-Cytochrome P-450 cam mono oxygenase (cam C gene) by PCR and gene probe
Source: Springerplus. 2013 Apr 17;2(1):170. doi: 10.1186/2193-1801-2-170 (PMC3647104; doi:10.1186/2193-1801-2-170)

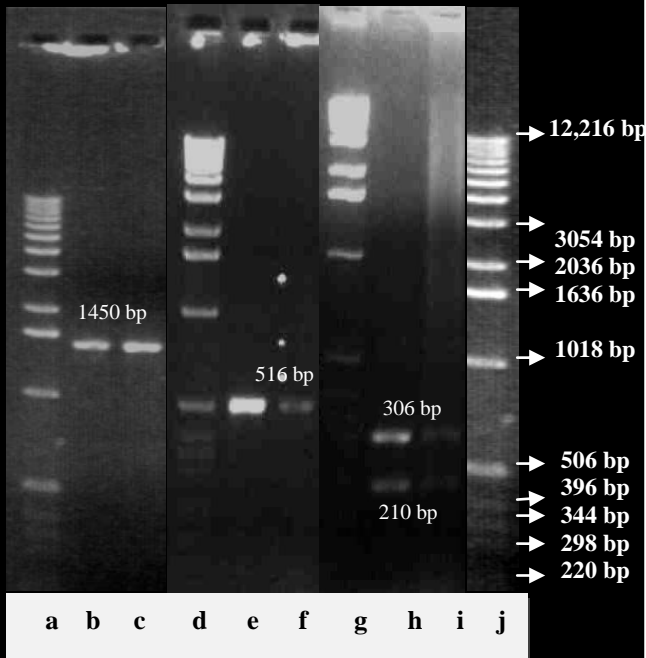

Supplement: Supplementary file 1 — Authors’ original file for figure 1 [file 40064_2013_238_MOESM1_ESM.pdf]

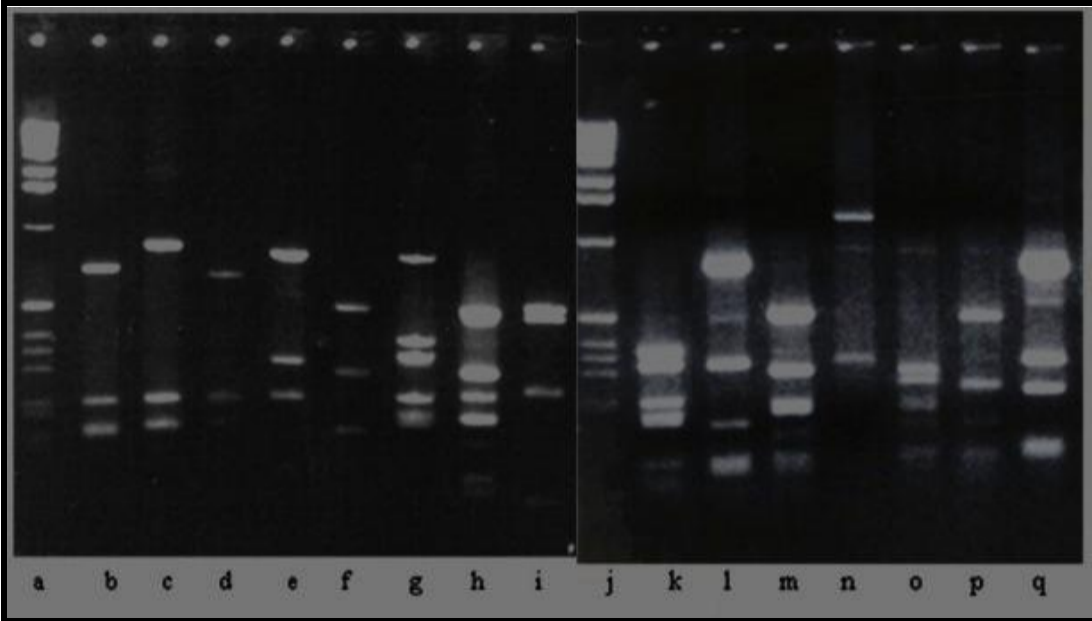

Supplement: Supplementary file 2 — Authors’ original file for figure 2 [file 40064_2013_238_MOESM2_ESM.pdf]

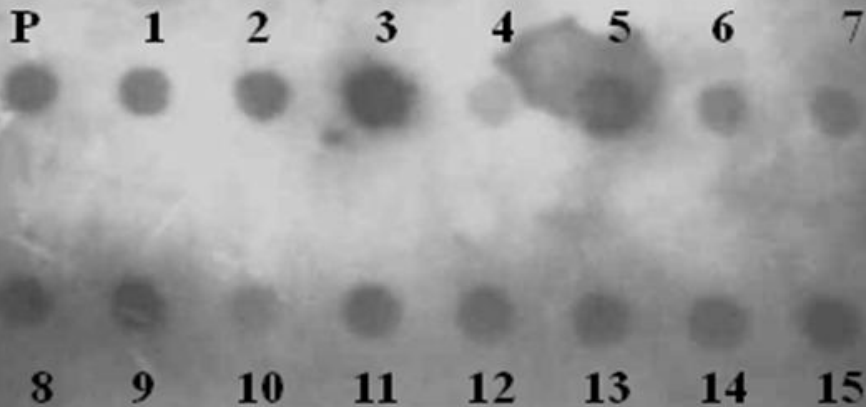

Supplement: Supplementary file 4 — Authors’ original file for figure 4 [file 40064_2013_238_MOESM4_ESM.pdf]

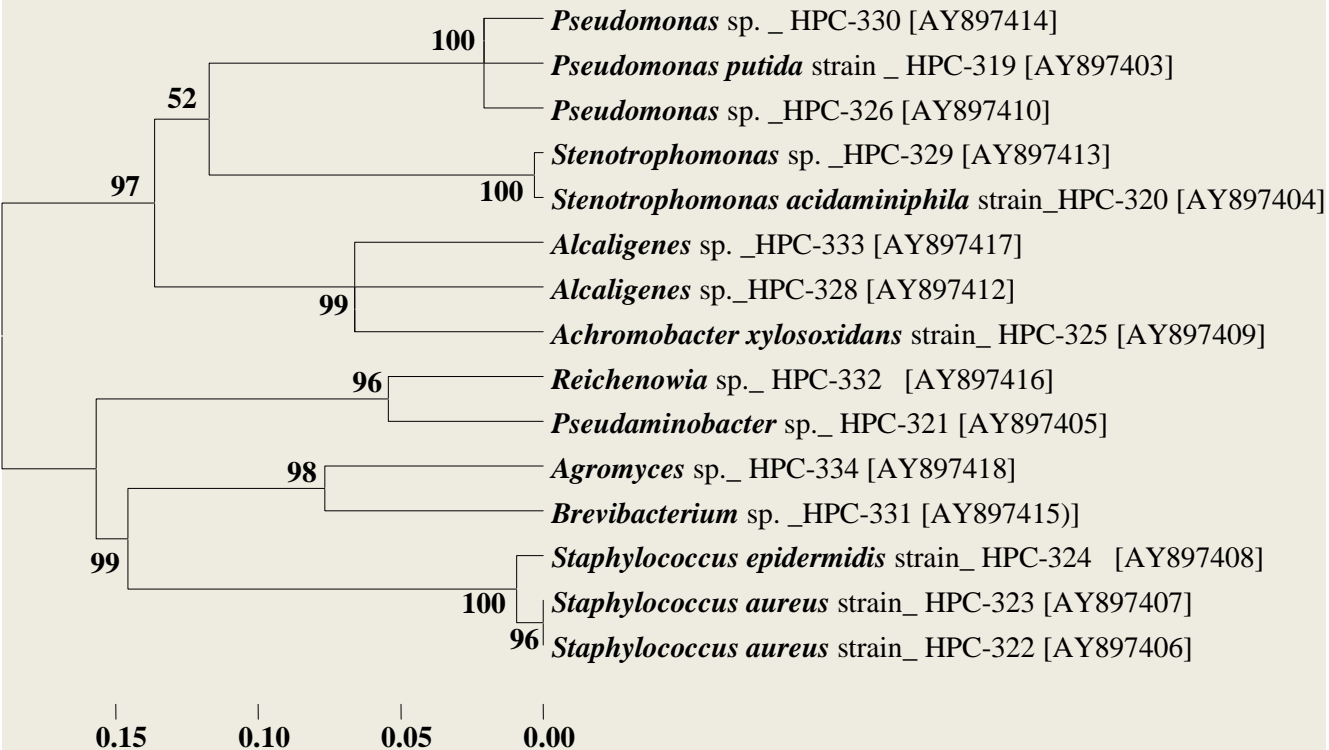

Supplement: Supplementary file 5 — Authors’ original file for figure 5 [file 40064_2013_238_MOESM5_ESM.pdf]
