# Supplementary material for: Molecular Characterization of camphor utilizing bacterial isolates from refinery sludge and detection of target loci-Cytochrome P-450 cam mono oxygenase (cam C gene) by PCR and gene probe
Source: Springerplus. 2013 Apr 17;2(1):170. doi: 10.1186/2193-1801-2-170 (PMC3647104; doi:10.1186/2193-1801-2-170)
Supplement: Supplementary file 3 — Authors’ original file for figure 3 [file 40064_2013_238_MOESM3_ESM.pdf]

atgacgactgaaaccatacaaagcaacgccaatcttgcccctctgccaccccatgtgccagagcacctg
gtattcgacttcgacatgtacaatccgtcgaatctgtctgccggcgtgcaggaggcctgggcagttctg
caagaatcaaacgtaccggatctggtgtggactcgctgcaacggcggacactggatcgccactcgcggc
caactgatc**cgtgaggcctatgaagattaccg**ccactttccagcgagtgcccgttcatccctcgtgaa
gccggcgaagcctacgacttcattcccacctcgatggatccgcccgagcagcgccagtttcgtgcgctg
gccaaccaagtggttggcatgccggtggtggataagctggagaaccggatccaggagctggcctgctcg
ctgatcgagagcctgcgcccgcaaggacagtgcaacttcaccgaggactacgccgaacccttcccgata
cgcatcttcatgctgctcgcaggtctaccggaagaagatatcccgcacttgaaatacctaacggatcag
atgacccgtccggatggcagcatgaccttcgcagaggccaaggaggcgctctacgactatctgataccg
atcatcgagcaacgcaggcagaagccgggaaccgacgctatcagcatcgttgccaacggccaggtcaat
gggcgaccgatcaccagtga**cgaagccaagaggatgtgtggc**ctgttactggtcggcggcctggatacg
gtggtcaatttcctcagcttcagcatggagttcctggccaaaagcccggagcatcgccaggagctgatc
gagcgtcccgagcgtattccagccgcttgcgaggaactactccggcgcttctcgctggttgccgatggc
cgcatcctcacctccgattacgagtttcatggcgtgcaactgaagaaaggtgaccagatcctgctaccg
cagatgctgtctggcctggatgagcgcgaaaacgcctgcccgatgcacgtcgacttcagtcgccaaaag
gtttcacacaccacctttggccacggcagccatctgtgccttggccagcacctggcccgccgggaaatc
atcgtcaccctcaaggaatggctgaccaggattcctgacttctccattgccccgggtgcccagattcag
cacaagagcggcatcgtcagcggcgtgcaggcactccctctggtctgggatccggcgactaccaaagcg
gtataa
